# The Role of Prophylactic Gastrectomy in Gastric Adenocarcinoma and Proximal Polyposis of the Stomach: A Systematic Review

**DOI:** 10.3390/jcm14072522

**Published:** 2025-04-07

**Authors:** Cosmina Fugărețu, Valeriu Marin Șurlin, Catalin Misarca, Daniela Marinescu, Stefan Patrascu, Sandu Ramboiu, Radu Petre, Victor Dan Eugen Strâmbu, Michael Schenker

**Affiliations:** 1 Doctoral School, Department of Surgery, University of Medicine and Pharmacy of Craiova, 200349 Craiova, Romania; comanescu_cosmina@yahoo.com (C.F.); vsurlin@gmail.com (V.M.Ș.); 2 1st General Surgery Department, Brașov County Emergency Clinical Hospital, 500326 Brașov, Romania; 3 Faculty of General Medicine Brașov, Transilvania University, 500036 Brașov, Romania; 4 1st General Surgery Department, Emergency Hospital of Craiova, 200642 Craiova, Romania; stef.patrascu@gmail.com (S.P.); sandu_r@yahoo.com (S.R.); 5 Faculty of General Medicine Craiova, University of Medicine and Pharmacy of Craiova, 200642 Craiova, Romania; mike_schenker@yahoo.com; 6 Faculty of Medicine, University of Medicine and Pharmacy “Carol Davila” Bucharest, 013346 Bucharest, Romania; drradupetru@yahoo.com (R.P.); drstrambu@yahoo.com (V.D.E.S.); 7 Surgery Clinic, “Carol Davila” Nephrology Hospital Bucharest, 013345 Bucharest, Romania; 8 Oncology Department, Emergency Hospital of Craiova, 200642 Craiova, Romania

**Keywords:** gastric adenocarcinoma and proximal polyposis of the stomach, prophylactic gastrectomy, gastric cancer, hereditary gastric cancer

## Abstract

**Background/Objectives**: Gastric adenocarcinoma and proximal polyposis of the stomach (GAPPS) is a recently discovered autosomal dominant transmission disease. Patients with this condition have a higher risk of developing gastric cancer. There are numerous questions regarding the natural history of this condition, as well as concerning the diagnostic and therapeutic management of these patients. In this systematic review, we aimed to examine the current literature to determine the role of prophylactic gastrectomy in patients diagnosed with gastric adenocarcinoma and proximal polyposis of the stomach. Additional outcomes are Helicobacter pylori (HP) infection, treatment with proton pump inhibitors (PPI), and colonoscopic examination and abdominal imaging examination, as they are important factors in the therapeutic decision. **Methods**: We performed a systematic review of the articles published in PubMed and Google Scholar, according to the PRISMA 2020 criteria. **Results**: We obtained 24 studies that included 83 patients diagnosed with GAPPS, of which 42 underwent prophylactic gastrectomy, 24 benefited from endoscopic follow-up, and 17 were diagnosed with gastric cancer at the first gastroscopic examination. In the prophylactic gastrectomy specimens, malignant gastric disease was confirmed in 10% of cases. GAPPS has been diagnosed more frequently in women. **Conclusions**: So far, the specialized literature includes a limited number of patients diagnosed with GAPPS. There are also no guidelines yet for the diagnosis and treatment of these patients. Prophylactic gastrectomy or endoscopic surveillance are the only options for patients diagnosed with GAPPS without gastric cancer at the initial examination. For prophylactic gastrectomy, the robotic and laparoscopic approach was preferred. For establishing appropriate lymphadenectomy in prophylactic gastrectomy, future research on gastrectomy specimens is necessary. Most of the included studies were deficient in terms of postoperative follow-up of patients. Thus, we consider it useful to include these patients in a single database. For a comprehensive examination of these and making an appropriate therapeutic decision, we consider it necessary to perform a colonoscopic evaluation, take abdominal imaging, and determine the Helicobacter pylori infection status.

## 1. Introduction

The incidence of gastric cancer has significantly decreased in recent times; however, it continues to be diagnosed in 990,000 people worldwide each year, and approximately 738,000 people die from this disease globally each year [1].

The familial aggregation of gastric cancer is encountered in 10% of cases, and only 1 to 3% of patients with gastric cancer are carriers of a germline mutation responsible for the onset of the disease [2]. Also, so far, three major hereditary syndromes are known to lead to the development of malignant gastric diseases, namely hereditary diffuse gastric cancer (HDGC), familial intestinal gastric cancer (FIGC), and adenocarcinoma and proximal polyposis of the stomach (GAPPS) [3].

It is known that familial adenomatous polyposis (FAP) is an autosomal dominant disorder characterized by the presence of pathogenic mutations in the Adenomatous polyposis coli (APC) gene. Patients with this condition develop numerous adenomatous polyps that progress to colorectal cancer [4]. It has also been found that approximately 70% of these individuals exhibit extraintestinal manifestations, leading to the introduction of a series of subcategories of the disease such as Gardner Syndrome, Turcot Syndrome, or GAPPS. Today, these conditions are no longer considered subcategories of familial polyposis but are included in a spectrum of diseases characterized by a specific mutation of the APC gene [4].

GAPPS is an autosomal dominant condition with incomplete penetrance, first discovered in 2011 in three families from the USA, Canada, and Australia [5].

Right now, the prevalence of this condition remains unknown. Nevertheless, there are authors who estimate that in the future it could reach up to 1% due to the improvement of diagnostic methods [6].

The diagnosis of GAPPS is based on Worthley’s criteria, which are clinico-endoscopic criteria presented in Table 1 [5,6]. In the case of individuals who meet the clinical criteria listed below, genetic testing is indicated to identify point mutations in the 1B promoter of the APC gene [7].

Meeting these criteria easily allows for differential diagnosis with the other two genetic syndromes mentioned above. Thus, HDGC is not characterized by the presence of gastric polyposis; it is determined by a complete inactivation of the CDH1 gene, resulting in reduced or even absent expression of E-cadherin, which leads to an increased risk of developing diffuse gastric cancer or lobular breast cancer (3).

On the other hand, for the diagnosis of FIGC, the Amsterdam criteria are used, and no specific genetic cause has been identified so far (3).

In 2016, it was discovered that affected individuals exhibit a mutation in the 1B promoter of the APC gene [8]. The mutations identified at the level of the 1B promoter of the APC gene are c. −191 T > C, c. −192 A > G, and c. −195 A > C [8]. Recently, the mutation c. 191 T > G has also been described. This was discovered in a Hispanic minor patient and a 35-year-old Asian man [9,10].

The presence of these mutations leads to the disruption of the YY1 transcription factor binding site, resulting in decreased transcription of the 1B promoter. At the level of the colon, isoform 1A can replace the function of the defective form 1B, but at the level of the stomach, this is not possible because isoform 1A is naturally permanently methylated [8].

Patients with GAPPS are asymptomatic for a long period of time. They may sometimes exhibit symptoms of dyspepsia, loss of appetite, or iron deficiency anemia [6].

Patients diagnosed with GAPPS have an increased risk of developing gastric adenocarcinoma, but the exact risk of malignancy is not yet known, and there is no consensus regarding their management [11].

Prophylactic gastrectomy is the surgical procedure that involves the removal of the stomach with the aim of reducing the risk of developing gastric cancer to zero. This intervention is recommended for patients at high risk of developing gastric cancer.

We reviewed the current literature to determine the role of prophylactic gastrectomy in patients with GAPPS, specifically when this intervention is indicated, what is the best approach, what are its benefits and drawbacks, and what are the non-surgical alternatives for managing this condition. Additional outcomes that we consider important in the diagnosis and treatment of this pathology are the status of Helicobacter pylori (HP) infection, treatment with proton pump inhibitors (PPI), colonoscopic examination, and imaging of the abdomen.

## 2. Materials and Methods

### 2.1. Protocol and Registration

This systematic review was conducted following the Preferred Reporting Items for Systematic Reviews and Meta-Analyses (PRISMA) 2020 criteria.

The protocol for this systematic review was registered on INPLASY (INPLASY2024100112).

### 2.2. Search Strategies

For the completion of this systematic review, we conducted a thorough search of the specialized literature available in the following databases: Medline via PubMed and Google Scholar. The following keywords were used: “gastric adenocarcinoma and proximal gastric polyposis-GAPPS”, “hereditary gastric cancer”, “prophylactic gastrectomy”, “endoscopy”, “gastric tumor”. These keywords were used in various combinations to find eligible studies. We manually checked the bibliographies of the articles included in order to identify other relevant studies.

### 2.3. Criteria for Inclusion and Exclusion

Inclusion criteria:

- Studies that included patients diagnosed with GAPPS who benefited from prophylactic gastrectomy and patients who did not benefit from this intervention. From the second category, we have patients with endoscopic follow-up, therapeutic gastrectomy, or patients with advanced gastric cancer and GAPPS who benefited only from chemotherapy regardless of their age;
- Original research articles and reviews, case reports, conference abstracts, and images;
- The language of the study’s publication, which was not to be a reason for exclusion.

The following criteria were used to exclude studies:

- Studies in which the diagnosis of GAPPS is not specified;
- Studies that included patients who underwent prophylactic gastrectomy due to the increased risk of HDGC or FIGC;
- Studies that included patients with prophylactic gastrectomy performed due to the increased risk of gastric cancer within other familial cancer syndromes, such as hereditary nonpolyposis colorectal cancer (HNPCC)-Lynch syndrome; Li-Fraumeni syndrome (LFS); FAP; and Peutz-Jeghers syndrome.

### 2.4. Data Extraction, Synthesis and Analysis

Two independent authors performed data extraction using a Microsoft Excel form (version 2502). The following data of interest were obtained from each study: the name of the first author, the type of study, the year of publication, the country of affiliation of the authors, the nationality of the patients included in the study, the definitive establishment of the GAPPS diagnosis according to the diagnostic criteria, the type of mutation in promoter 1B of the APC gene, the presence of a family history of GAPPS or other cancers, the number of cases included in the study, the ages and sexes of the patients, the number of cases that underwent prophylactic gastrectomy, the number of cases that only received follow-up, and the number of cases diagnosed with GAPPS and gastric cancer. The result of the upper digestive endoscopy, colonoscopy, and imaging examinations-CT were also noted for each case. Another point of interest was the presence of HP infection and the history of PPI treatment.

In the case of patients who underwent prophylactic gastrectomy, information such as the duration between diagnosis and prophylactic gastrectomy, the type of gastrectomy performed, and the surgical approach used were also extracted. A series of characteristics of the surgical intervention were also noted, such as the extension of lymphadenectomy and the method of restoring digestive continuity. The mode, the duration of postoperative follow-up, and postoperative treatment were other parameters of interest. The histopathological results presented in each article were also analyzed.

Disagreements among investigating authors were resolved through discussions and consultation with other authors of the present study.

Due to the small number of identified studies as well as their heterogeneity, we did not perform a meta-analysis. Thus, we presented the results of the identified studies in the form of tables and graphs and discussed their most important aspects.

To assess the risk of bias, we used The Newcastle–Ottawa Scale (NOS), which has a maximum score of 9.

## 3. Results

**Figure 1:**
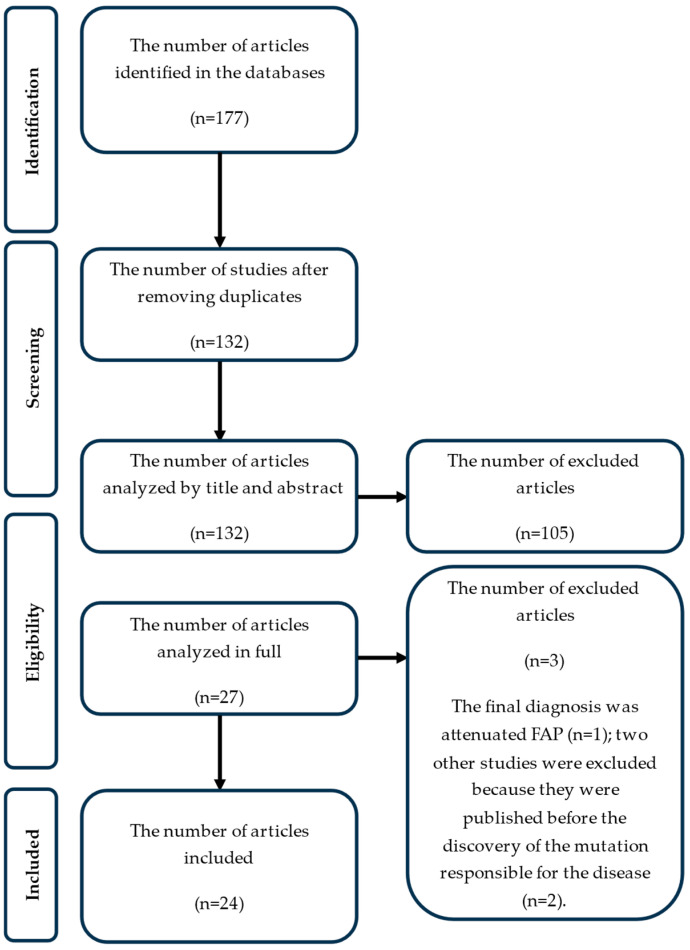
PRISMA flowchart.

After searching the specified databases, 177 studies were identified, and 45 duplicate studies were identified and removed. After removing duplicates, the number of studies was 132. These were analyzed by title and abstract, and 105 were excluded because they did not contain relevant information for the current research. In the end, 27 papers remained that were read and analyzed in their entirety. Among these, 3 were excluded:

- One study because the final diagnosis was attenuated FAP (*n* = 1);
- Two other studies because they were published before the discovery of the mutation responsible for the disease (*n* = 2),

A total of 24 studies that met the inclusion criteria were identified. In Figure 1, the PRISMA flow diagram is presented.

### 3.1. Characteristics of the Studies and Demographic Data of the Enrolled Patients

The articles included in this study were published between January 2016 and July 2024.

In Table 2, all included studies are presented along with their most important characteristics, such as the name of the first author, the year and place of publication, the type of study, and the number of patients included in each study. Other information that we considered relevant and which is presented in Table 3 included the ages and sexes of the included patients, the method of diagnosis through esophagogastroduodenoscopy (EGD), the type of genetic mutation of the APC gene promoter 1B, the performance of other diagnostic explorations such as colonoscopy or imaging examinations, and the history of treatment with PPI or the presence of HP infection. We noted the number of cases where prophylactic gastrectomy was performed on those where this intervention was not carried out. Patients who did not benefit from prophylactic gastrectomy were further divided into those who received endoscopic surveillance, therapeutic gastrectomy, or were diagnosed with advanced gastric cancer and received only chemotherapy. The presence of a family history of GAPPS or other cancers was specified for each included patient, as well as their nationality. We also looked into the duration between the establishment of the diagnosis and the performance of prophylactic gastrectomy. Regarding the operative aspects, we noted the method of performing the gastrectomy, the extent of the lymphadenectomy, the method of restoring digestive continuity, and the duration of the surgical intervention.

Among the postoperative aspects, we noted the histopathological results of the resection specimens, the method, and the duration of postoperative follow-up, the last two parameters being, unfortunately, the least frequently specified in the identified studies.

Most of the included studies were case reports. The number of cases presented in the studied articles was 83, and in 42 of these, prophylactic gastrectomy was performed; 8 patients required therapeutic gastrectomy. In 24 cases diagnosed with GAPPS, periodic endoscopic follow-up was chosen, and 9 patients diagnosed with gastric cancer and GAPPS received only oncological treatment (Table 3).

The quality scores of the studies ranged from 2 to 7, reflecting a high to moderate risk of bias due most frequently to the small number of cases and deficiencies in control selection.

The average age of patients who underwent prophylactic gastrectomy was 42 years, with the caveat that in 14 cases the age of the patients was not specified [16,19,26]. The youngest patient diagnosed with GAPPS was only 8 years old and underwent a prophylactic gastrectomy at 10 years old [23]. The oldest patient who underwent a prophylactic gastrectomy identified in our study was 71 years old [27].

The average age of patients who did not undergo prophylactic gastrectomy was 44.8 years, with the youngest being only 22 years old, and in four cases the age of the subjects was not specified [16,29,30].

Regarding the gender distribution of individuals with GAPSS who underwent prophylactic gastrectomy, women were the most numerous, with a total of 17, followed by 12 men, and in two studies that included 5 and 8 patients, respectively, their sex was not specified [19,26].

Women represented the highest percentage among patients who required a therapeutic gastrectomy (Table 3). Additionally, out of the total number of patients diagnosed with GAPPS and gastric cancer, 85% were women.

**Figure 2:**
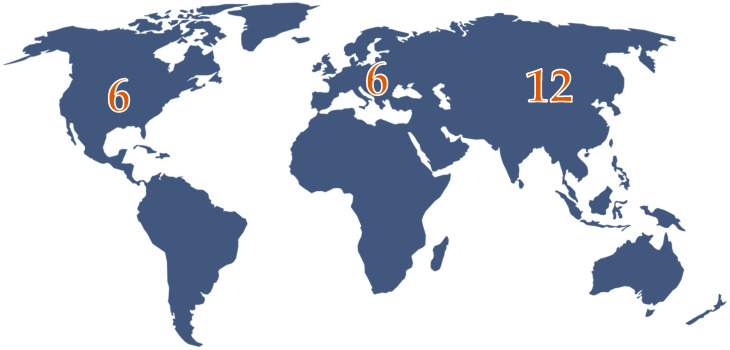
Global distribution of articles included in the study.

The largest number of articles was published in Asia, namely 12; 6 articles come from Europe, and another 6 articles come from the USA (Figure 2). However, a Czech study published in 2019 included the largest number of patients diagnosed with GAPPS, namely 24 patients, while most other studies included presentations of 1–8 cases [16].

In the end, from the 83 patients included in the study, 45 of the patients diagnosed with GAPPS are European, 32 Asian, and 6 American.

### 3.2. Clinical-Diagnostic Aspects of the Enrolled Patients

Out of the total of 83 patients in the included studies, in 70 cases, EGD was performed. EGD was not performed in the case of a single patient carrying the c. −191 T > C mutation due to his advanced age of 92 years [16].

In over 80% of the cases that benefited from prophylactic gastrectomy, EGD was performed. In 8 cases that underwent robotic prophylactic gastrectomy, the performance of EGD is not specified, but it is noted that the diagnosis of GAPPS was established (Table 3) [26].

In 3 cases, where the presence of the 1B promoter mutation of the APC gene (c. −191 T > C) was confirmed, EGD did not reveal the presence of gastric polyps, and these patients only benefited from endoscopic monitoring (Table 3) [16,30].

The performance of a colonoscopy is not specified in more than half of the cases diagnosed with GAPPS. A percentage of 20.48% of patients with GAPPS did not have pathological changes in the colon; however, the presence of polyps was confirmed in 13.25% of cases, and only in one case was the presence of sigmoid diverticula specified. This information is available in Table 3.

Among the patients who underwent prophylactic gastrectomy, 21.42% did not show pathological changes upon colonoscopic examination, while 19.04% of the patients had the presence of colonic polyps detected.

Regarding the history of treatment with PPI, in more than half of the studied cases, this is not specified. Furthermore, the duration of this treatment is mentioned in only two cases, being 6 months and 10 years, respectively [16]. A quarter of the patients who underwent therapeutic gastrectomy and those diagnosed with advanced gastric cancer without surgical benefit had a history of PPI treatment (Table 3).

Additionally, 16.66% of patients with prophylactic gastrectomy had a history of PPI treatment; however, in most cases, it was not specified whether they received PPI or not (Table 3).

The presence of HP infection was confirmed in 3 cases [25,30]. In 2 of these cases, it was eradicated 6 years before, but both patients were diagnosed with gastric cancer and underwent therapeutic gastrectomy [30].

The most frequent mutation of promoter 1B found in our studies was c. −191 T > C. The rarest mutations encountered were c. −191 T > G, which was specified in two cases, and c. 195 A > C, which was discovered in a patient who was only 8 years old and underwent prophylactic gastrectomy at just 10 years old [9,10,23]. In 30 cases included in 10 studies, the exact type of the 1B promoter mutation of the APC gene was not mentioned.

Imaging of patients diagnosed with GAPPS is mentioned in less than 25% of all cases and was not specified in any of the cases that underwent endoscopic follow-up. All cases that underwent therapeutic gastrectomy had a normal imaging appearance on the preoperative computer tomography (CT). Only in 6 cases, which underwent prophylactic gastrectomy, the normal appearance of the preoperative CT scan is specified [11,18,23,25].

All patients diagnosed with GAPPS in our study had a family history of gastric cancer, GAPPS, or even other cancers such as lung cancer, prostate cancer, colorectal cancer, leukemia, or hepatocellular carcinoma, and most of the included studies presented a series of related cases [9,24,25,29].

In our study, we identified a total of 21 patients diagnosed with GAPPS and gastric cancer, of which 9 cases specified their deaths.

### 3.3. Operative and Postoperative Aspects of Patients with GAPPS Who Underwent Prophylactic Gastrectomy

Regarding the duration between the establishment of the GAPPS diagnosis and the performance of prophylactic gastrectomy, this was not specified in over 75% of cases. In 4 cases, prophylactic gastrectomy was performed more than 10 years after the endoscopic detection of proximal gastric polyposis, while in the other 7 cases, it varied between 1 and 4 years [12,13,16,23,24,29]. The longest interval between the endoscopic detection of proximal gastric polyposis and the performance of prophylactic gastrectomy was over 40 years [27].

**Figure 3:**
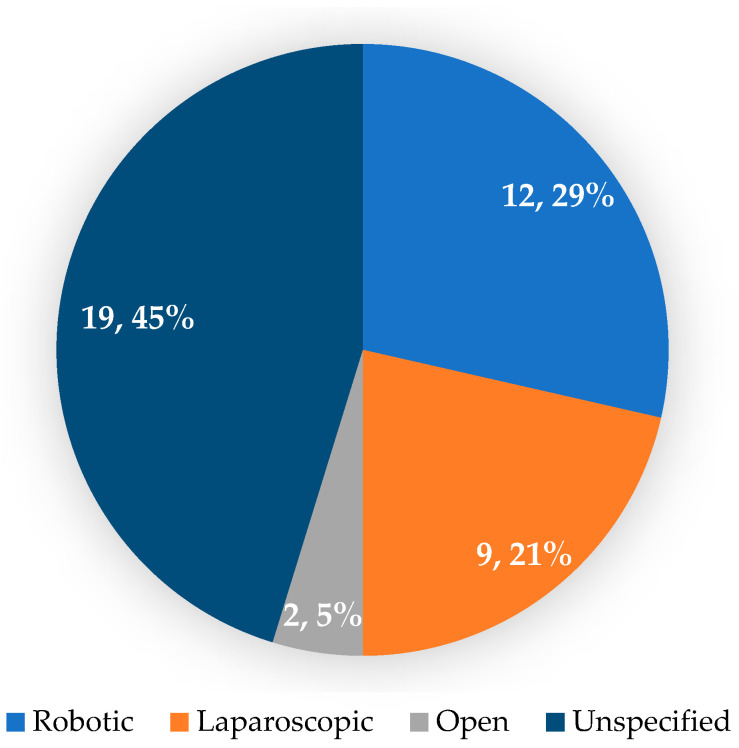
The surgical approach for patients with GAPPS and prophylactic gastrectomy.

In almost half of the cases that underwent prophylactic gastrectomy, the surgical approach was not indicated; most prophylactic gastrectomies performed for GAPPS were robotic (Figure 3). Most patients underwent a total prophylactic gastrectomy, and only one study presents the case of a patient diagnosed with GAPPS who underwent a robotic upper polar gastrectomy followed by a reconstruction through a Roux-en-Y esojejunostomy and a jejunogastric anastomosis that allows for subsequent endoscopic examination of the remaining stomach [32].

**Figure 4:**
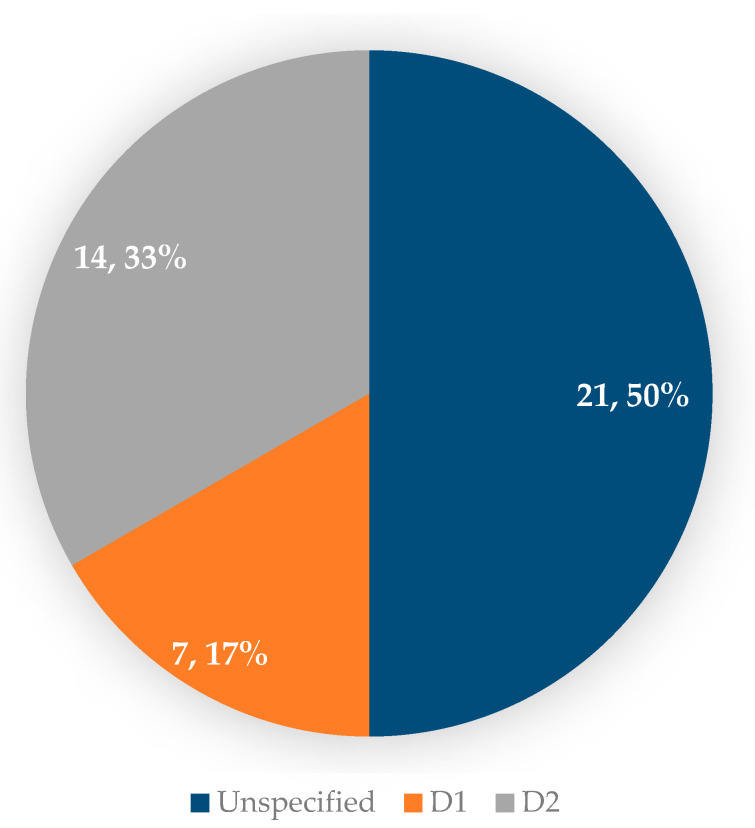
The extension of lymphadenectomy in patients with GAPPS and prophylactic gastrectomy.

The extent of lymphadenectomy was not specified in half of the cases, and the number of patients who underwent D2 lymphadenectomy was double compared to those who underwent D1 lymphadenectomy (Figure 4).

In 16 cases of total prophylactic gastrectomy, the method of restoring digestive continuity through esojejunostomy in a Roux-en-Y configuration is specified.

Only in two studies, which included a total of 11 patients and in which all prophylactic gastrectomies were performed robotically, the duration of the surgeries was specified, and this varied between 273 and 337 min [25,26].

Analyzing the histopathological results of the prophylactic gastrectomy specimens, we found that the presence of dysplasia was recorded in 15 cases, with 4 of these being high-grade, and in another 4 cases, the presence of malignant cells was confirmed [11,12,13,16,24,27,32]. In 2 of the cases diagnosed with postoperative gastric cancer, prophylactic gastrectomy was performed 2 and 4 years, respectively, after the diagnosis of GAPPS, and both patients were in stage IA [12].

The duration of postoperative follow-up for GAPPS patients who underwent prophylactic gastrectomy was specified in only two studies, and this varied between 2 and 36 months [11,25].

In a single study, the type of postoperative follow-up was also specified, and this consisted of performing an endoscopy every 6 months and an abdominal CT scan 1 year after the surgical intervention [11]. The same study also specifies the postoperative weight loss of patients as well as the postoperative treatment with Fe, Ca, and vit B12 [11].

Only one study mentions the resumption of professional activity one month after prophylactic gastrectomy for GAPPS [18].

## 4. Discussion

GAPPS is an autosomal-dominant disorder with an allele frequency of 0.001 and a penetrance of 80%; however, experts anticipate an increase in prevalence, primarily due to the enhancement of diagnostic techniques [6,8].

Most of the studies included in our article comprise case series from the same family. Therefore, genetic testing of relatives is necessary, but it is difficult to establish an optimal age for starting endoscopic surveillance, considering that the youngest patient diagnosed with GAPPS was an 8-year-old boy [23].

In the pediatric population, gastric cancer is found in a percentage ranging from 0.05% to 0.10% [33]. It seems that this pathology is detected in the pediatric population at more advanced stages of the disease compared to the adult population; the survival of these patients compared to the adult population diagnosed at the same stage is similar [34].

In our study, we identified 2 minor patients, under 18 years old, both of whom underwent prophylactic gastrectomy at 10 and 16 years of age, respectively, and the histopathological examination revealed the presence of high-grade dysplasia in the 10-year-old patient [9,23].

There are individuals for whom genetic testing confirms the presence of a mutation at the 1B promoter level of the APC gene, yet EGD does not reveal the characteristic features of GAPPS [8].

Less than 4% of the patients enrolled in our study did not present polyposis upon endoscopic examination, even though they are mutation carriers.

There are authors who recommend that endoscopic monitoring should begin at the age of 15 for patients with a family history and mutations in the promoter 1B region of the APC gene, or even earlier in the presence of a dyspeptic syndrome, with the procedure to be repeated every 5 years if no polyps are identified in the proximal stomach [6]. On the other hand, other authors recommend performing an upper gastrointestinal endoscopy annually along with an abdominal ultrasound, even in the absence of gastric polyps [16].

If individuals have a family history of GAPPS but are not carriers of the aforementioned mutations, some studies recommend endoscopic examination starting at the age of 18, and if proximal gastric polyps are absent, follow-up is no longer necessary [6].

It has been observed that the onset of gastric polyposis in patients carrying genetic mutations of promoter 1B can vary from 13 to 16 years, but the youngest patient diagnosed with proximal gastric polyposis was only 8 years old [16,23]. It seems that the age of onset of gastric polyps in patients with GAPPS can be influenced, in addition to the genetic pattern, by lifestyle or environmental factors [16].

Regarding the sex ratio of patients diagnosed with GAPPS, it has been in favor of women, thus raising the question of whether hormonal profile may play a role in the clinical manifestation of this pathology.

Until now, there is no consensus regarding the management of patients diagnosed with GAPPS [11]. Furthermore, the natural history of this condition is not yet fully understood, nor are the mechanisms of progression to gastric cancer, but some studies have estimated the risk of gastric cancer occurrence in patients carrying the c. −191 T > C mutation to be 25% [7,16].

In the included articles, a case was identified where the diagnosis of gastric cancer with distant metastases was made only 10 months after the diagnosis of GAPPS [22]. On the other hand, another case where the presence of gastric polyps was endoscopically confirmed 41 years previously did not show signs of malignancy on the prophylactic gastrectomy specimen; however, both parents and this person’s cousin were diagnosed with gastric cancer in the past [27]. The presence of these significant variations between the detection of proximal gastric polyposis and the appearance of malignant gastric lesions raises even more issues in the management of GAPPS.

Endoscopic surveillance alongside prophylactic gastrectomy represents the main options for the therapeutic management of this condition.

For the early diagnosis of precancerous epithelial gastric lesions, it is recommended to perform an endoscopy every 3 years for individuals with non-extensive precancerous conditions or first-degree relatives diagnosed with gastric cancer [35].

Performing an endoscopic ultrasound can assess whether there is submucosal invasion or adjacent adenopathy, which draws attention to the malignant transformation of some polypoid lesions [36].

It seems that endoscopic examination following the Systematic Screening Protocol for the stomach (SSP) is superior to standard endoscopy in the early detection of neoplastic lesions in patients at risk of hereditary gastric cancer, including GAPPS [37]. This protocol is standardized and involves pre-examination preparation with the administration of mucolytics, as well as the administration of antiperistaltic agents such as Buscopan or glucagon [38].

The use of artificial intelligence (AI), specifically deep learning (DL), seems promising in the early detection of premalignant lesions and early gastric cancer and in assessing the depth of malignant invasion [39]. Thus, DL is a branch of artificial intelligence capable of using numerous databases to learn to recognize certain lesions, functioning similarly to a neural network [40]. In some cases, the use of endoscopic systems that employ AI had results comparable to those of experienced human endoscopists, and there were even situations where the latter are outperformed by these systems with integrated AI [41]. Thus, is it possible to monitor patients diagnosed with GAPPS using endoscopic examination with the help of AI? Can this postpone prophylactic gastrectomy, especially in pediatric cases where the surgical intervention may have significant consequences on psychosomatic development?

On the other hand, endoscopic surveillance with submucosal excision of suspicious lesions can constitute a management approach for this condition, while being aware of the risk of gastric cancer developing between examination periods [28].

AI can also be used in creating the family tree. Thus, this computerized system based on machine learning and DL techniques can be useful to oncogeneticists both for the digitalization and construction of the family tree and for the automatic prediction of genetic predisposition risk [42].

Periodic endoscopic biopsy of polypoid lesions cannot accurately assess the risk of gastric cancer; therefore, patients without significant comorbidities are indicated for prophylactic gastrectomy [11]. Additionally, considering the large number of polyps, it is difficult to determine how many tissue fragments are necessary at each digestive endoscopy for effective monitoring of these patients [23].

If in the case of patients with CDH1 gene mutations, performing prophylactic gastrectomy is indicated starting at the age of 20, and there are thus several studies that have evaluated the subsequent quality of life of these patients; the same cannot be said for patients with GAPPS [43,44].

Although patients who undergo prophylactic gastrectomy are usually younger and theoretically at lower risk of postoperative complications compared to patients requiring gastrectomy for gastric cancer, there are studies that have found the rate of reinterventions for complications to be similar [45].

Updated European guidelines for clinical management of GAPPS published in May 2024 recommend considering prophylactic total gastrectomy due to the risk of gastric cancer in cases of high-grade dysplasia or progressive polyposis, but there is still insufficient evidence regarding the age at which prophylactic gastrectomy is indicated, and thus the decision must be individualized [46].

Some authors recommend performing prophylactic gastrectomy in patients diagnosed with GAPPS without endoscopically identified dysplasia between the ages of 30–35 or 5 years earlier than the age at which the youngest relative was diagnosed with malignant gastric disease, while other authors consider that the risk of gastric cancer occurrence in patients with GAPPS may be overestimated in the current context of underdiagnosis of this syndrome [6,47].

Preservation of the gastric antrum in the absence of polyps at this level may be accompanied by some benefits for the patient in terms of quality of life and nutritional status. The association of an end-to-side gastrojejunostomy may allow for endoscopic examination of the remaining stomach. So far, we have identified a single study in which proximal gastric resection was chosen [32].

Regarding the surgical approach, laparoscopic gastrectomy performed for gastric cancer seems to have similar short-term oncological benefits to the classical approach, to which, of course, the advantages of the minimally invasive approach are added [48]. At Oslo University Hospital, since 2015, the preferred approach for gastrectomy has been laparoscopic [26,49].

Robotic gastrectomy is increasingly gaining ground in the surgical treatment of gastric cancer, with studies showing that the robotic approach may be superior to the laparoscopic approach in terms of lymphatic dissection accuracy and patient hospitalization duration [50]. A lower rate of pancreatic fistulas has been observed in patients who underwent robotic gastrectomy compared to those who underwent laparoscopic gastrectomy. [51]. The superior ergonomics encountered in the robotic approach, along with the greater precision of the operator, are other advantages of robotic surgery, while the higher cost of this approach is currently considered the main disadvantage [25].

In the included articles, prophylactic gastrectomy was most frequently performed using robotic and laparoscopic approaches.

Another important aspect is the extent of lymphadenectomy in the prophylactic gastrectomy of patients with GAPPS, which was not specified in almost half of the cases included in the study. There are authors who opted for a D2 lymphadenectomy, considering the risk of postoperative diagnosis of gastric cancer [19,26]. We must specify that only one patient diagnosed with GAPPS and gastric cancer post-prophylactic gastrectomy had lymph node invasion, most being diagnosed at early stages [16]. Thus, we consider that the decision regarding the extent of lymphadenectomy accompanying prophylactic gastrectomy for GAPPS should be made individually for each patient. An unjustified extension of lymphadenectomy in a young patient diagnosed with GAPPS who is undergoing prophylactic rather than therapeutic gastrectomy can increase the morbidity and mortality of the patient without a certainty of benefits. We also believe that more studies are needed to precisely determine the extent of lymphadenectomy in prophylactic gastrectomy for GAPPS.

Regarding the long-term postoperative management of patients diagnosed with GAPPS who have undergone prophylactic total gastrectomy, this is lacking in almost all included studies.

There are numerous consequences of prophylactic gastrectomy; these are physical, psychological, social, and economic [43]. From the first category, we can mention weight loss, which can go up to 20% of body weight, eating disorders, dumping syndrome and diarrhea, anemia, osteoporosis, etc. [43]. Eating disorders are frequently encountered, so the majority of patients have had to reduce the amount of food consumed at a meal and increase the frequency of meals. Dumping syndrome and diarrhea are experienced by the majority of patients; however, significant improvement in these symptoms has been observed over time. Additionally, over half of the patients who undergo prophylactic gastrectomy experience fluctuations in blood glucose levels that persist for years after the surgical intervention [44].

Also, the absorption disorders of Fe, vitamin B12, and calcium should not be overlooked, which is why most patients require lifelong multivitamin supplements [44].

If, at least theoretically, the young age of individuals undergoing prophylactic gastrectomy, along with the absence of associated diseases, can correlate with a lower risk of immediate postoperative complications, metabolic and nutritional disorders can influence long-term growth and development.

On the other hand, it has been observed that younger patients experience a faster postoperative recovery, and approximately one year postoperatively, their quality of life can be similar to that existing preoperatively; however, this is not the case in all instances [45].

As far as the psychosocial consequences of prophylactic gastrectomy performed on patients at risk of HDGC, a decrease in anxiety related to cancer risk was observed, but in some cases, there were recorded impairments in relational abilities as well as a decline in finances, necessitating even professional reorientation [43]. Another study that included 54 patients who underwent prophylactic gastrectomy for the CDH1 gene mutation and were followed on average for over 4 years concluded that this intervention is safe when performed in reference centers, and the postoperative quality of life is good despite the presence of persistent nutritional disorders [44]. Additionally, pregnancies in women who have undergone prophylactic gastrectomy have also been reported in the literature [52].

Regarding the long-term postoperative management of patients who have undergone prophylactic total gastrectomy, it must be appropriate considering that these patients are usually young and have a high life expectancy.

Another important aspect is that the survival of patients diagnosed with GAPPS and stage IV gastric cancer was lower compared to the average survival of patients with sporadic forms at the same stage [53].

The main advantages and disadvantages of endoscopic surveillance and prophylactic gastrectomy are summarized in Table 4.

Regarding the long-term administration of PPIs, this can lead to the appearance of gastric fundic polyps through glandular dilation and oxyntic cell hyperplasia; however, this process is reversible after stopping the treatment [54]. Furthermore, the polyps are fewer in number and are also found in the gastric antrum, unlike the polyps in GAPPS [6]. There is no correlation between the level of hypergastrinemia and the presence of gastric polyps in patients who have undergone long-term treatment with PPIs [55]. On the other hand, there are authors who recommend discontinuing PPI treatment in patients with GAPPS and considering the administration of H2 antagonists to these patients if they have more than 20 polyps or if the polyps are larger than 10 mm. [6].

A particular aspect of GAPPS is the low rate of HP infection in these patients. There are two cases cited in the literature where the acquisition of HP infection resulted in the regression of gastric fundic polyps; however, this observation was made approximately 10 years before the recognition and definition of GAPPS [56]. Additionally, situations have been identified where gastric polyps appeared after the eradication of HP infection and regressed with the onset of reinfection with this bacterium [57]. It is still unknown whether HP infection in patients with GAPPS is less common due to an unfavorable gastric environment for the mentioned bacterial infection or if it plays a protective role in the development of gastric polyps [6].

An article published in 2024 presents the case of a 44-year-old patient in whom gastric polypoid formations appeared after the eradication of HP infection, thus confirming the diagnosis of GAPPS five years after the treatment of atrophic gastritis. The patient was diagnosed with stage IA gastric adenocarcinoma and underwent a total robotic gastrectomy with D1 lymphadenectomy [30]. It cannot be determined with certainty whether the malignant gastric disease in this case appeared as a result of atrophic gastritis induced by HP infection or GAPPS, with the possibility of both risk factors being associated [30]. On the other hand, the brother of the patient presented above, who carries the same genetic mutation, was diagnosed with a gastric ulcer and HP infection but without polyposis on endoscopic examination [30]. Thus, could HP infection complicate the diagnosis of GAPPS in the context of observing the absence of polyps in patients carrying a mutation in the 1B promoter, and especially how should we proceed with these patients?

So far, the status of HP infection does not influence the clinical management of patients with GAPPS [58].

A first step that we consider necessary is testing all patients carrying the mutation for GAPPS and for HP infection, especially in the context of the absence of gastric polyps at the initial endoscopy. Also, we consider that endoscopic monitoring at shorter intervals, every 6 months, is necessary in the case of eradicating this infection in patients diagnosed with GAPPS and without gastric polyposis at the initial examination. Thus, we consider that endoscopic follow-up at short intervals, every 6 months, is necessary for patients diagnosed with GAPPS without polyps at the initial endoscopic examination and in whom HP infection has been eradicated.

On the other hand, it seems that individuals diagnosed with GAPPS also have a higher risk of developing extragastric tumors such as colon cancer, pancreatic cancer, leukemia, lung cancer, brain cancer, thyroid cancer, skin cancer, or prostate cancer [1,14,29,54]. A clinical trial aimed at evaluating the concomitant presence of colonic polyps in patients with GAPPS concluded that these individuals more frequently present polyps in the colon compared to other family members without GAPPS. It is presumed that colonic polyps appear when promoter 1A does not fully compensate at the colonic level for the dysfunction of promoter 1B [58,59]. Therefore, we advocate for the systematic performance of colonoscopies and at least one abdominopelvic imaging examination for patients diagnosed with GAPPS.

Regarding the histopathological aspect of polyps in GAPPS, it is not characteristic enough to allow for diagnosis without genetic testing, just as in other syndromes where gastric polyps are encountered, such as juvenile polyposis or Peutz-Jeghers syndrome; the histopathological aspect has a diagnostic accuracy of less than 50% [58]. Several histopathological features more frequently encountered in GAPPS are areas of aberrant hyperproliferation of oxyntic glands at the level of gastric pits (HPAPs), thus making the progression from adenoma to dysplasia to cancer feasible [60].

The most frequent mutation of the 1B promoter of the APC gene found in the patients included in the study was c. −191 T > C. In the pediatric population, the c. −195 A > C mutation was more frequently identified [23,58]. The c. −191 T > G mutation was identified in two cases: in a 35-year-old Asian patient and in a 16-year-old minor patient [9,10]. The first patient was diagnosed with gastric cancer and underwent therapeutic gastrectomy, and the minor patient, who exhibited all the endoscopic characteristics of GAPPS, underwent prophylactic gastrectomy [9,10]. Thus, this rare genetic variant draws our attention to the importance of continued research and thorough examination of these patients. Unfortunately, in over half of the cases that underwent prophylactic gastrectomy, although the presence of the 1B promoter mutation of the APC gene is specified, it is not accurately detailed. We consider the establishment of a unique database useful to allow the systematization of the variants of the 1B promoter mutation of the APC gene.

The most common gaps we identified in the included studies were the lack of a decision-making algorithm and a protocol for the postoperative follow-up of patients who underwent prophylactic gastrectomy.

## 5. Conclusions and Future Perspectives

GAPPS is an autosomal dominant genetic condition that has malignant neoplastic potential, and the specialized literature currently presents only a limited number of cases diagnosed worldwide with GAPPS.

Endoscopic identification of proximal gastric polyposis necessarily requires genetic testing for the presence of mutations in the 1B promoter of the APC gene.

Although there is currently no standard protocol for the diagnosis and treatment of patients with GAPPS, it is very useful to determine the status of HP infection in all cases. After the eradication of HP infection in patients with 1B promoter mutations but without gastric polyps, it is very important to repeat EGD due to the risk of subsequent development of gastric polyps. Future studies are also necessary to accurately determine the role of HP infection in patients with GAPPS.

We recommend complementing the endoscopic examination with a colonoscopy and at least one abdominal imaging examination due to the higher risk of the presence of colonic polyps or other neoplasms.

The decision to pursue endoscopic surveillance or perform a prophylactic gastrectomy must be made individually for each case.

The presence of dysplastic areas in gastric polyps requires the performance of prophylactic gastrectomy.

Prophylactic gastrectomy is the only option for reducing the risk of gastric cancer to zero in patients diagnosed with GAPPS. Both the laparoscopic and robotic approaches are the best options for performing prophylactic gastrectomy. Regarding the extent of lymphadenectomy, more studies are needed to evaluate the stage of cases identified with gastric cancer in prophylactic gastrectomy specimens. Regarding the postoperative management of these patients, the existing protocols for patients who have undergone prophylactic gastrectomy for hereditary diffuse gastric cancer can be successfully applied.

Right now, it is not known why this pathology has been diagnosed more frequently in women. Thus, studying the role that sex hormones may play in the development of proximal gastric polyposis could constitute a new research direction.

The early diagnosis of patients, their inclusion in a dedicated database, and the development of a treatment guide are very important for reducing the gastric cancer mortality of these patients. Additionally, a multidisciplinary approach that includes a complex team consisting of a gastroenterologist, geneticist, surgeon, pathologist, nutritionist, and psychologist can substantially contribute to the successful management of GAPPS cases.

## Figures and Tables

**Table 1:** Worthley’s criteria for the diagnosis of gastric adenocarcinoma and proximal polyposis of the stomach (GAPPS) [5].

Inclusion Criteria	Exclusion Criteria
Endoscopic identification of numerous polyps located in the gastric fundus and body, excluding the presence of duodenal and colorectal polyps [5]	Patient with gastric polyps with long-term treatment with proton pump inhibitors—it is recommended to repeat the endoscopy after discontinuing the treatment [5]
Very large number of polyps, over 100, or over 30 polyps in the case of a close relative of another case [5]	Patient with other criteria that allow the diagnosis of hereditary gastric polyposis within other diseases [5]
Polypi located at the level of the gastric fundus presenting dysplasia [5]	
A family member diagnosed with gastric fundic polyposis or gastric adenocarcinoma [5]	
Demonstration of autosomal dominant genetic transmission [5]	

**Table 2:** Summary of the included studies.

First Author Name and References (REF)	Study Design	Year of Publication of the Study	The Place Where the Study Was Conducted	The Number of Patients Included
Repak, R. et al. [12]	case report and review	2016	Europe—Czech Republic	4
Beer, A. et al. [13]	case report	2017	Europe—Austria	1
Yano, M. et al. [14]	case report	2018	Asia—Japan	1
Mitsui, Y.et al. [15]	case report	2018	Asia—Japan	3
Foretova, L. et al. [16]	original article	2019	Europe—Czech Republic	24
Kunovsky, L. et al. [17]	case report	2019	Europe—Czech Republic	2
Anderson, A. et al. [18]	case report	2019	USA—Minnesota	1
Mala, T. et al. [19]	case report and review	2020	Europe—Norway	5
Kanemitsu, K. et al. [20]	case report	2020	Asia—Japan	4
Ako, S. et al. [21]	image	2020	Asia—Japan	3
Matsumoto, C. et al. [22]	case report	2021	Asia—Japan	3
Roberts, A.G. et al. [9]	case report	2021	USA—Florida	1
Grossman, A. et al. [23]	case report	2021	USA—NY	1
Powers, J. et al. [24]	image	2021	USA—Pennsylvania	1
Salami, A.C. et al. [11]	case series and review	2022	USA—Philadelphia	2
Iwakawa, Y. et al. [25]	case report	2022	Asia—Japan	4
Mala, T. et al. [26]	research article	2022	Europe—Norway	8
Sakuma, T. et al. [27]	case report and review	2023	Asia—Japan	6
Saito, Y. et al. [28]	e-videos	2023	Asia—Japan	1
Iwamuro, M.et al. [29]	case report	2023	Asia—Japan	3
Okamoto, K. et al. [30]	case report	2024	Asia—Japan	2
Hirai, R. et al. [31]	case report	2024	Asia—Japan	1 (3 cases presented, also appear in Ako, S.)
Ishida, A. et al. [10]	case report	2024	Asia—Japan	1
Judge, S.J. et al. [32]	case report	2024	USA—California	1

**Table 3:** The main characteristics of the patients included in the study.

	Patients with Prophylactic Gastrectomy	Patients Without Prophylactic Gastrectomy			Total
		Patients with Therapeutic Gastrectomy	Patients with Endoscopic Follow-Up	Patients with Gastric Cancer Receiving Only Oncological Treatment	
Patients diagnosed with GAPPS	42 (50.60%)	8 (9.63%)	24 (28.91%)	9 (10.84%)	83 (100%)
Patients average age (years)	42 years (8–71 years)	43.1 years (24–69 years)	45.3 years (22–92 years)	45.5 years (26–64 years)	43 years
Gender					
F	17 (40.47%)	7 (87.50%)	18 (75%)	7 (77.77%)	49 (59.03%)
M	12 (28.57%)	1 (12.50%)	6 (25%)	2 (22.27%)	21 (25.30%)
Unspecified	13 (30.95%)	0 (0%)	0 (0%)	0 (0%)	13 (15.66%)
EGD					
YES polyps	34 (80.95%)	8 (100%)	19 (79.16%)	9 (100%)	70 (84.33%)
Normal	0(0%)	0 (0%)	3 (12.50%)	0 (0%)	3 (3.61%)
NOT performed	0(0%)	0 (0%)	1 (4.16%)	0 (0%)	1 (1.20%)
Unspecified	8 (19.04%)	0 (0%)	1 (4.16%)	0 (0%)	9 (10.84%)
Colonoscopy					
Normal appearance	9 (21.42%)	6 (75%)	1 (4.16%)	1 (11.11%)	17 (20.48%)
Presence of polyps	8 (19.04%)	1 (12.5%)	0 (0%)	2 (22.22%)	11 (13.25%)
Unspecified	24 (57.14%)	1 (12.5%)	23 (95.83%)	5 (55.55%)	53 (63.85%)
NOT performed	0(0%)	0 (0%)	0 (0%)	1 (11.11%)	1 (1.20%)
Other pathologies	1 (2.38%)	0 (0%)	0 (0%)	0 (0%)	1 (1.20%)
PPI treatment					
YES	7 (16.66%)	2 (25%)	2 (8.33%)	2 (22.22%)	13 (15.66%)
NO	8 (19.04%)	5 (62.5%)	3 (12.50%)	7 (77.77%)	23 (27.71%)
Unspecified	27 (64.28%)	1 (12.5%)	19 (79.16%)	0 (0%)	47 (56.62%)
HP infection					
YES	0 (0%)	2 (25%)	1 (4.16%)	0 (0%)	3 (3.61%)
NO	7 (16.66%)	6 (75%)	5 (20.83%)	2 (22.22%)	20 (24.09%)
Unspecified	35 (83.33%)	0 (0%)	18 (75%)	7 (77.77%)	60 (72.28%)
Genetic mutation					
c. 191 T > C	14 (33.33%)	6 (75%)	16 (66.66%)	7 (77.77%)	43 (51.80%)
c. 191 T > G	1 (2.38%)	1 (12.5%)	0 (0%)	0 (0%)	2 (2.40%)
c. 195 A > C	1 (2.38%)	0 (0%)	0 (0%)	0 (0%)	1 (1.20%)
c. 195 A > G	3 (7.14%)	0 (0%)	3 (12.5%)	0 (0%)	6 (7.22%)
c. −30,217 T > C	1 (2.38%)	0 (0%)	0 (0%)	0 (0%)	1 (1.20%)
non-specific	22 (52.38%)	1 (12.5%)	5 (20.83%)	2 (22.22%)	30 (36.14%)
Imagistics CT					
Normal	6 (14.28%)	8 (100%)	0 (0%)	0 (0%)	14 (16.86%)
Pathologic	1 (2.38%)	0 (0%)	0 (0%)	3 (33.33%)	4 (4.81%)
Unspecified	35 (83.33%)	0 (0%)	24 (100%)	6 (66.66%)	65 (78.31%)

**Table 4:** The main advantages and disadvantages of endoscopic surveillance and prophylactic gastrectomy at patients with gastric adenocarcinoma and proximal polyposis of the stomach (GAPPS).

	Esophagogastroduodenoscopy	Prophylactic Gastrectomy
Advantages	Allows visualization of polypoid lesions and collection of serial biopsies for the early identification of dysplasia and malignant transformation [11,23]; Allows submucosal excision of lesions suspected of malignancy [28]; It can be repeated; It does not significantly change the quality of life.	Eliminates the risk of gastric cancer.
Disadvantages	There are no recommendations regarding the age at which endoscopic surveillance should be initiated [6,16]; Due to multiple polypoid lesions, areas of malignant transformation may be overlooked; this risk can be mitigated by following the Systematic Screening Protocol. [23,28,37]; Gastric cancer lesions can develop between examinations. [11,28].	It is irreversible; It is accompanied by postoperative risks such as anastomotic leak [45]; It can have long-term nutritional and psychosocial consequences [43]; It is difficult to determine the age at which it is indicated [6,46]; In the case of female individuals, we must also consider the patient’s desire to have a pregnancy. [52]

## Data Availability

The authors confirm that the data supporting the findings of this study are available within the article.
